# Gonadoblastoma in Turner syndrome with puberty delay: A case report and literature review

**DOI:** 10.1002/mgg3.2300

**Published:** 2023-10-12

**Authors:** Wei Shen, Ya Li

**Affiliations:** ^1^ Department of Obstetrics and Gynecology Tongji Hospital, Tongji Medical College, Huazhong University of Science and Technology Wuhan China

**Keywords:** case report, gonadoblastoma, malignancy risk, puberty induction, Turner syndrome, Y chromosome material

## Abstract

**Background:**

Y chromosome material stands as an independent risk determinant for the onset of gonadoblastoma (GB) and subsequent gonadal germ cell tumours in individuals with Turner syndrome (TS). However, the delayed and underestimated identification of Y chromosome material through karyotyping within primary care settings exacerbates the intricacies of managing these patients over the long term.

**Methods:**

We present a case involving TS accompanied by Y chromosome material, wherein puberty delay and GB were identified during prophylactic gonadectomy. Subsequently, we delve into the literature to explore the GB‐related malignancy risk in TS patients with Y chromosome material, the incidence of Y chromosome presence in TS patients using methodologies beyond routine chromosomal testing, and the diagnosis and treatment of puberty delay in TS patients, all based on our case.

**Results:**

A spectrum of more sensitive molecular techniques, including polymerase chain reaction (PCR) and fluorescence *in situ* hybridisation, effectively augments the detection of Y chromosome material alongside karyotyping. In addition to gonadectomy, the implementation of appropriate oestrogen therapy and a holistic, multidisciplinary approach to care can enhance the quality of life, while mitigating the long‐term morbidity and mortality risks for TS patients harbouring Y chromosome material.

**Conclusions:**

Beyond gonadectomy, adopting a multifaceted approach the Y chromosome material detection, prompt initiation of puberty, tailored oestrogen therapy, and coordinated multidisciplinary management significantly contributes to the comprehensive health oversight of TS patients with Y chromosome material.

## Graphical abstract

Drawing upon a case report of a TS patient with Y chromosome material and a comprehensive literature review, we underscore the paramount significance of employing multiple Y chromosome material detection techniques, timely initiation of puberty, appropriate oestrogen therapy, and holistic multidisciplinary management in addition to gonadectomy for individuals with TS and Y chromosome material.

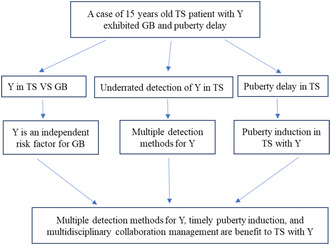

## 1 INTRODUCTION

Turner syndrome (TS) is a prevalent genetic condition arising from the partial or complete loss of the second X chromosome in phenotypic females. Its occurrence is noted in approximately 1 in 2000–2500 live female births (Gravholt, 2005; Gravholt et al., 2017). Individuals with TS commonly manifest traits such as diminished stature, deferred pubertal onset, ovarian inadequacy, as well as cardiac and renal irregularities, sensorineural hearing impairment, ocular complications, thyroid anomalies, metabolic syndrome, inflammatory bowel disease, and neurocognitive challenges (Gravholt et al., 2019; Huang et al., 2021). The presence of a normal or partially deleted Y chromosome is discerned in 4%–61% of TS patients contingent upon the methodology employed, thereby signifying a 10%–30% risk of developing gonadoblastoma (GB) (Dabrowski et al., 2020). GB, classified as a borderline tumour arising from sex cord and germ cells, is intrinsically linked with gonadal dysgenesis. Approximately 40% of these occurrences exhibit bilaterality, and up to 35% of cases can potentially evolve into malignant tumours, including seminoma and dysgerminoma (Looijenga, Hersmus, et al., 2007). Consequently, the counsel of preventive gonadal resection once diagnosis is established stands as a pivotal recommendation. Furthermore, meticulous preoperative evaluation, judicious intraoperative scrutiny, and discernment of histopathological attributes are imperative for achieving a precise diagnosis. Subsequently, an organised regimen of follow‐up and comprehensive multidisciplinary clinical oversight is equally vital. This context presents a case of TS marked by the presence of a Y chromosome and pubertal delay, wherein GB was unveiled upon prophylactic gonadectomy. The ensuing discourse incorporates a review of pertinent literature encompassing the GB‐associated malignancy risk in TS patients with Y chromosome material, the occurrence of Y chromosome material in TS patients via both methodological and routine chromosome testing, along with the delineation and management of pubertal delay in TS patients. Furthermore, it contemplates the lessons drawn from our case in terms of management outcomes.

## 2 CASE PRESENTATION

A 15‐year‐old phenotypic female initially presented at the gynaecology clinic due to primary amenorrhoea. A chromosomal analysis indicated 45XO/46XY, prompting a referral for gonadectomy. Her medical history encompassed short stature and delayed puberty, with no record of migraines, motor and learning impairments, or surgical interventions. No familial history of malignancies was evident. Her 9‐year‐old brother exhibited normal stature and cognitive development within the peer group, while her father's height measured 180 cm, and her mother displayed short stature (145 cm). On physical examination, she displayed an unremarkable appearance, notable for her diminutive height (141 cm) and slight breast development (B3). Notably absent were specific Turner syndrome (TS) indicators, such as a short webbed neck, disproportional upper and lower body segments, or multiple nevi.

**FIGURE 1:**
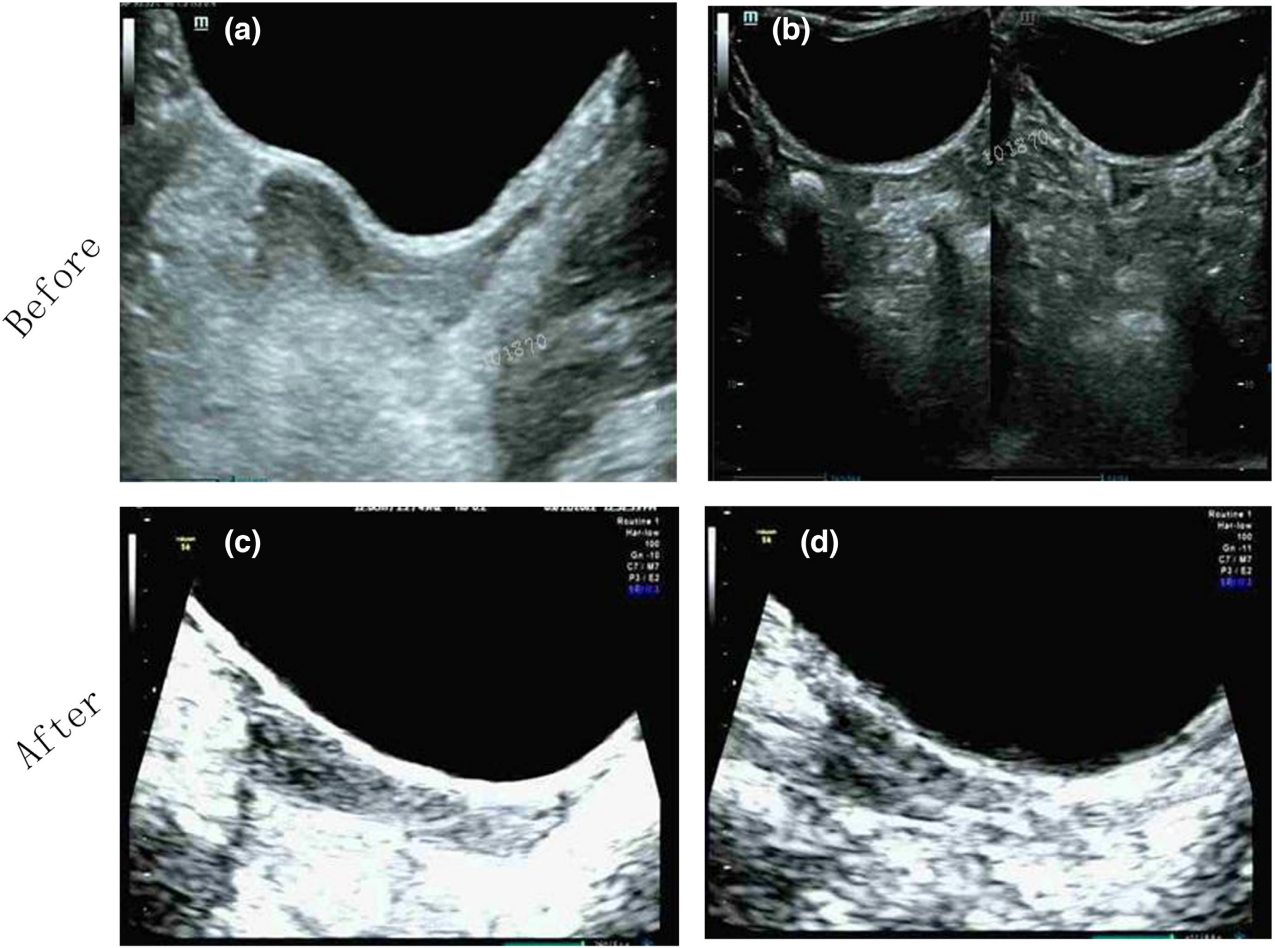
Pelvic ultrasound before and after laparoscopic bilateral gonadectomy. (a) Pelvic ultrasound image prior to laparoscopic bilateral gonadectomy depicting the uterus. (b) Pelvic ultrasound image prior to laparoscopic bilateral gonadectomy revealing both gonads. (c) Pelvic ultrasound image post laparoscopic bilateral gonadectomy and after 3 months of puberty induction, displaying the uterus. (d) The endometrium appears linear following 3 months of puberty induction.

Given the augmented risk of germ cell malignancies linked to the presence of Y chromosome material, tumour markers (human chorionic gonadotropin, alpha‐fetoprotein, and lactate dehydrogenase) were evaluated and showed negativity, albeit with a slightly elevated alpha‐fetoprotein. Hormone assessments unveiled hypergonadotropic hypogonadism, evidenced by elevated FSH levels (80.13 mIU/mL) and decreased estradiol (16.77 pg/dL). An ultrasound was conducted to evaluate genitourinary anatomy and screen for potential tumours. No abnormalities were noted in her renal, liver, cardiac, and major vascular structures. Pelvic ultrasonography revealed a diminutive uterus (2.11.22.3 cm) with a body‐to‐cervix ratio of 1:1 (Figure 1a). The left ovary was not visualised, whereas the right adnexa displayed abnormalities suggestive of a right ovary (Figure 1b).

**FIGURE 2:**
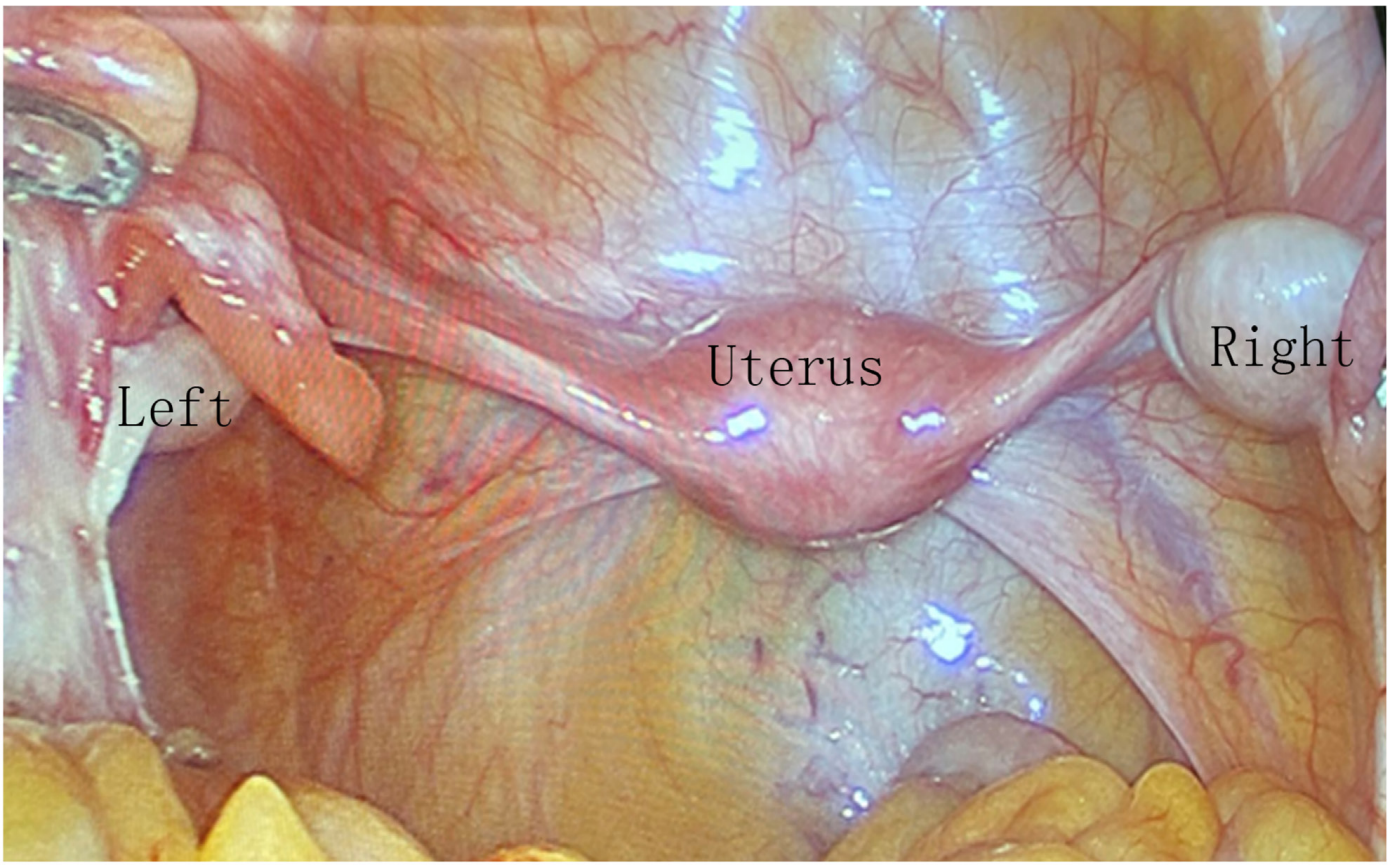
Pelvic assessment by laparoscopy. Laparoscopy unveiled the left gonad in a streak formation (left), while the right gonad exhibited a normal “ovarian” appearance (right). Both fallopian tubes displayed robust development, although the uterus appeared to be in an infantile state (uterus).

Both the patient and her family underwent counselling regarding the multisystem health implications of TS, the premalignant nature of gonadoblastoma (GB), the malignancy risk associated with TS patients possessing Y chromosome material, and the repercussions of pubertal delay in TS patients. Subsequently, the patient and her parents provided consent for prophylactic laparoscopic bilateral gonadectomy. Laparoscopy revealed a streak gonad on the left side, while the right ovary exhibited normal appearance (Figure 2). Both ovaries and fallopian tubes were excised, while the uterus was preserved. Surgical pathology examinations unveiled GB in both gonads, with scant hypoplastic testicular tissue detected in the left gonad under microscopic analysis. The bilateral fallopian tubes exhibited no pathological abnormalities. The patient was discharged according to the predetermined plan and experienced an uncomplicated recovery. To initiate puberty, she commenced a low‐dose oestrogen regimen (estradiol valerate, 0.5 mg/day). Three months post‐surgery, a plain and enhanced abdominal CT scan was conducted, revealing no indications of pelvic or abdominal masses or lymph node involvement. Additionally, increased uterine volume (2.41.02.4) was discerned through ultrasound (Figure 1c,d).

## 3 DISCUSSION

Turner syndrome (TS) is associated with various conditions, including impaired social cognitive processing, executive function deficits, and social dysfunction. The well‐being of TS patients is compromised, leading to a decrease in their quality of life and a reduction of approximately 13–15 years in their average life expectancy (Aversa et al., 2021; Gravholt et al., 2017; Sawyer et al., 2018). The presence of hypogonadism necessitates that roughly 90% of TS patients undergo oestrogen therapy to either initiate or complete their pubertal development. This imperative is particularly heightened for TS patients with Y chromosome material, who require both gonadectomy to mitigate germ‐cell tumour risks and timely pubertal induction (Gravholt et al., 2017; Sawyer et al., 2018). While karyotyping detects only 6% of TS patients with Y chromosome material, technological advancements have unveiled a substantially higher prevalence of Y chromosome material.

Gonadoblastoma (GB) is an uncommon borderline tumour speculated to emanate from the rudimentary gonad in certain cases of disorder of sex development (DSD). It is typified by an amalgamation of large germ cells akin to seminoma and smaller sex cord stromal cells, including immature Sertoli and granulosa cells (Hersmus et al., 2017). Literature outlining the natural history of GB is sparse, as it is generally encountered during gonadectomy (Morin, Peard, & Saltzman, 2020). A prevailing theory posits that GB originates from germ cells initially following the female developmental trajectory (ovarian), yet becoming arrested in primordial follicles due to failure to conclude the prophase of mitosis (Cools et al., 2006). Classical GB is predominantly observed within dysgenetic gonads. In concurrence with our case, pathological analysis revealed GB in both gonads, where microscopic examination of the left gonad further illuminated a modest amount of hypoplastic testicular tissue. This finding underscores the potential of undifferentiated gonadal tissue to act as a precursor to GB.

Moreover, TS patients possessing Y chromosome material are classified within an intermediate risk category for the development of gonadal malignancies (Looijenga, Gillis, et al., 2007; Looijenga, Hersmus, et al., 2007). This classification is rooted in the association of gonadoblastoma on the Y‐chromosome (GBY) region with an escalated susceptibility to type II germ cell tumours/cancer (Du et al., 2014). Furthermore, deficient SOX9 expression within GB is linked to an increased vulnerability for malignant transformation (Kao et al., 2014).

In summation, GB assumes the role of the most prevalent precursor to malignant germ cell tumours in individuals grappling with disorders of sex development featuring a Y chromosome, in part or whole. Therein, the Y chromosome‐associated GB stands to undergo eventual transformation into invasive dysgerminoma or other malignant germ cell tumours (GCTs) including embryonic carcinoma, teratoma, yolk sac tumour, and choriocarcinoma, at an incidence range of 35%–60% (Armstrong et al., 2020; Kao et al., 2014; Looijenga, Hersmus, et al., 2007; Morin, Peard, & Saltzman, 2020; Morin & Saltzman, 2021; Scully, 1970). However, it is important to note that the diagnosis of GB primarily hinges on pathological assessment following gonadectomy. Neither conventional ultrasound nor MRI can definitively exclude neoplasms in gonadal dysgenesis (GD) patients with intra‐abdominal gonads (Ebert et al., 2018). As puberty marks a pinnacle period for GCT transformation (Morin, Peard, Vanadurongvan, et al., 2020), and adult GD patients harbouring the Y chromosome exhibit the highest GCT risk (Slowikowska‐Hilczer et al., 2020), the majority of TS patients with a Y chromosome, including cases akin to ours, typically undergo gonadectomy either prior to puberty or promptly subsequent to chromosomal diagnosis.

Prophylactic gonadectomy is exclusively administered to patients displaying Y chromosome components, although routine chromosome testing may overlook mosaic TS cases harbouring Y chromosome material, as well as those with GB. Karyotyping identifies a normal or structurally aberrant Y chromosome in 5%–12% of TS patients. Despite the apparent Y chromosome detection in TS patients, molecular studies such as polymerase chain reaction (PCR) and fluorescence *in situ* hybridization (FISH) (Akcan & Boduroglu, 2021; Bispo et al., 2014; Chen et al., 2021; Cortes‐Gutierrez et al., 2012; Silva‐Grecco et al., 2016) have heightened the identification of concealed Y chromosome material in TS patients, consequently elevating the Y‐chromosome sequence detection rate to approximately 60% (de Marqui et al., 2016). Furthermore, the risk of GB development among patients with cryptic Y chromosome material mirrors that of individuals possessing overt Y chromosome anomalies (Kwon et al., 2017).

Despite the potential for gonadal malignancies, the overall survival (OS) outcomes remain commendable. For those diagnosed with GB during prophylactic gonadectomy, the 5‐year OS and recurrence‐free survival (RFS) rates surpass 96%. Even those with a considerably diminished risk of germ cell tumours at the time of gonadectomy exhibit 5‐year OS and RFS rates of 87% and 82%, respectively (Morin, Peard, Vanadurongvan, et al., 2020). While deliberations regarding potential post‐pubertal surgery delay persist, a complete Y chromosome presents itself as the primary risk factor for GB, advocating for early gonadectomy (Karila et al., 2022). The discourse regarding the timing of gonadectomy notwithstanding, judicious contemplation is requisite for puberty induction and the long‐term management of persistent comorbidities.

Puberty, a transformative phase bridging childhood and adulthood, is recognised to commence between the ages of 7 and 13 years, spanning approximately a decade, and culminating between 18 and 20 years, signifying attainment of physical and sexual maturity along with reproductive capacity (Abreu & Kaiser, 2016; Sawyer et al., 2018).

The chronology of puberty closely adheres to a nearly normal distribution within the general population. This temporal trajectory is predominantly governed by genetic factors, though it is also influenced by variables such as weight, nutrition, psychosocial circumstances, and potential endocrine‐disrupting agents in the environment. Conditions linked to gonadal impairment can instigate delayed puberty (Seppa et al., 2021). While 20%–42% of TS girls might experience spontaneous puberty and menstruation (Bucerzan et al., 2017), the presence of hypergonadotropic hypogonadism characterises TS girls due to gonadal dysgenesis, particularly in cases featuring Y‐chromosome material. Such individuals are presumed to harbour nonfunctional gonads or may have undergone prophylactic gonadectomy. Notably, the majority of TS patients encounter variable extents of puberty delay, predominantly attributed to ovarian failure, as observed in our case. A retrospective cohort study underscored that approximately 70% of TS patients exhibited indications of ovarian failure, with the median onset at 11 years (Fitz et al., 2021). Consequently, hormone replacement therapy emerges as the principal intervention for managing puberty delay in TS patients. This therapeutic approach seeks to achieve breast development, uterine maturation, and an adolescent growth spurt congruent with physiological processes, while mitigating potential side effects (Butler & Purushothaman, 2020).

The most recent clinical practice guidelines for the management of girls and women afflicted with TS propose an initial regimen that commences with a conservative dosage of oestrogen (Gravholt et al., 2017). While the routine incorporation of exceedingly minimal oestrogen doses prior to puberty is not advocated, circumstances where elevated gonadotropin levels (serum FSH ≥ 10 U/L at age ≥ 10 years) or diminished AMH concentrations arise, necessitate oestrogen therapy (administered via injection, percutaneous, or oral means) commencing at the ages of 11–12 years. Subsequently, the dosage is incrementally augmented every 6 months, contingent upon individual responses and remaining growth potential. This stepwise augmentation mirrors typical adolescent processes, eventually culminating in an adult dose within 2–3 years. Introduction of progesterone is undertaken once breakthrough bleeding while on oestrogen therapy emerges, typically occurring 2–3 years later or upon ultrasonographic indications of endometrial maturation and thickness. The principal objectives of oestrogen administration encompass replicating the natural course of puberty in girls, optimising growth prospects, and mitigating associated risks. Delaying the initiation of oestrogen treatment could adversely impact uterine, osseous, and psychosocial dimensions of health (Aversa et al., 2021; Sawyer et al., 2018; Webber et al., 2016).

## 4 CONCLUSIONS

While the clinical landscape might indicate early infancy as the timeframe for diagnosis, several TS patients receive diagnoses during later childhood or even adolescence, precisely when pubertal development is conspicuously absent, and pubertal derailment becomes evident through primary amenorrhoea, akin to our case. Given that the presence of Y chromosome material operates as an autonomous risk determinant for GB and is intrinsically linked to an escalated susceptibility to type II germ cell tumours/cancer, the identification of Y chromosome material within TS cases significantly impacts patients’ long‐term well‐being and quality of life. The deployment of refined molecular techniques, encompassing PCR and FISH, furnishes heightened precision in Y chromosome material detection compared to conventional karyotyping. In tandem with gonadectomy for TS patients featuring Y chromosome material, the integration of oestrogen therapy and multidisciplinary collaborative care exerts substantial influence over enduring morbidity and mortality outcomes (Aversa et al., 2021; Gravholt et al., 2017; Sawyer et al., 2018).

## AUTHOR CONTRIBUTIONS

Wei Shen collected the case, literature the reviews and written the article, Ya Li audited the case and the total article.

## FUNDING INFORMATION

None.

## CONFLICT OF INTEREST STATEMENT

The authors have no relevant financial or non‐financial interests to disclose.

## ETHICS APPROVAL

This work has been carried out in accordance with the Declaration of Helsinki (2000) of the World Medical Association. This study was approved by the Ethics Committee of the Tongji Hospital of Huazhong University Tongji Medical College, and all participants provided written informed consent. All methods were carried out in accordance with relevant guidelines and regulations.

## CONSENT TO PARTICIPATE

Informed consent was obtained from all individual participants included in the study.

## PERMISSION TO REPRODUCE MATERIAL FROM OTHER SOURCES

Not applicable.

## CLINICAL TRIAL REGISTRATION

Not applicable.

## Data Availability

The data that support the findings of this study are available from the corresponding author upon reasonable request.
